# Minocycline inhibits glial proliferation in the H-Tx rat model of congenital hydrocephalus

**DOI:** 10.1186/1743-8454-7-7

**Published:** 2010-05-27

**Authors:** James P McAllister, Janet M Miller

**Affiliations:** 1 Department of Neurosurgery, Division of Pediatric Neurosurgery, Primary Children's Medical Center and the University of Utah, Salt Lake City, UT 84132, USA; 2 Department of Biology, Central Michigan University, Mt. Pleasant, MI 48859, USA

## Abstract

**Background:**

Reactive astrocytosis and microgliosis are important features of the pathophysiology of hydrocephalus, and persistent glial "scars" that form could exacerbate neuroinflammation, impair cerebral perfusion, impede neuronal regeneration, and alter biomechanical properties. The purpose of this study was to determine the efficacy of minocycline, an antibiotic known for its anti-inflammatory properties, to reduce gliosis in the H-Tx rat model of congenital hydrocephalus.

**Methods:**

Minocycline (45 mg/kg/day i.p. in 5% sucrose at a concentration of 5-10 mg/ml) was administered to hydrocephalic H-Tx rats from postnatal day 15 to day 21, when ventriculomegaly had reached moderate to severe stages. Treated animals were compared to age-matched non-hydrocephalic and untreated hydrocephalic littermates. The cerebral cortex (both gray matter laminae and white matter) was processed for immunohistochemistry (glial fibrillary acidic protein, GFAP, for astrocytes and ionized calcium binding adaptor molecule, Iba-1, for microglia) and analyzed by qualitative and quantitative light microscopy.

**Results:**

The mean number of GFAP-immunoreactive astrocytes was significantly higher in untreated hydrocephalic animals compared to both types of controls (*p *< 0.001). Minocycline treatment of hydrocephalic animals reduced the number of GFAP immunoreactive cells significantly (*p *< 0.001). Likewise, the mean number of Iba-1 immunoreactive microglia was significantly higher in untreated hydrocephalic animals compared to both types of controls (*p *< 0.001). Furthermore, no differences in the numbers of GFAP-positive astrocytes or Iba-1-positive microglia were noted between control animals receiving no minocycline and control animals receiving minocycline, suggesting that minocycline does not produce an effect under non-injury conditions. Additionally, in six out of nine regions sampled, hydrocephalic animals that received minocycline injections had significantly thicker cortices when compared to their untreated hydrocephalic littermates.

**Conclusions:**

Overall, these data suggest that minocycline treatment is effective in reducing the gliosis that accompanies hydrocephalus, and thus may provide an added benefit when used as a supplement to ventricular shunting.

## Background

Shunt failure continues to be one of the largest problems facing those who suffer with hydrocephalus. Despite the initial benefits of shunting, 40% of children with hydrocephalus undergo shunt revisions within the first year, and 50% will have a shunt revision within the first 2 years of insertion [1]. Shunts fail for numerous reasons; one likely cause is failure of the shunt valve to actuate properly due to the changing hydrostatic properties of a stiff, non-compliant brain [2]. This lack of compliance may be attributed to a proliferation of glial cells.

Gliosis is an important feature of the pathophysiology of hydrocephalus [3-10], and persistent glial "scars" that form in the shunted brain could promote neuroinflammation, impair cerebral perfusion, alter the blood-brain barrier, prevent repair of damaged neural tissue and impede neuronal plasticity, and change intracranial compliance which poses additional problems for optimal shunt function. We have shown previously that gliosis in the form of reactive astrocytosis and microgliosis increases significantly in the H-Tx rat model of congenital hydrocephalus [9,10]. These changes occur in both cortical and subcortical regions, and while ventricular shunting can reduce gliosis in this model, it does not restore glial parameters to normal levels.

Experimental and clinical studies have demonstrated that minocycline, a semi-synthetic tetracycline derivative, inhibits the proliferation of microglial cells after various types of experimental brain injuries and clinical disorders, and is an effective neuroprotective agent [11-17] (reviewed recently by Buller *et al *[18] and Hailer [19]). Minocycline has also been shown to be effective in reducing astrogliosis [13]. These promising results support the hypothesis that gliosis could be reduced or prevented during the progression of hydrocephalus by the administration of minocycline. This study is the first investigation of minocycline treatment in hydrocephalus-induced gliosis and therefore represents a promising new approach in the treatment of this condition.

## Methods

### Subjects

All procedures conducted in this study were approved by the Animal Care and Use Committee of Wayne State University. A colony of H-Tx rats, which develop congenital hydrocephalus due to a closure of their cerebral aqueduct between embryonic day 18 and postnatal day 5 [20-25], was maintained at Wayne State University. Hydrocephalus progresses rapidly in these animals, and by 21 days of age they become severely hydrocephalic. If these hydrocephalic animals are not treated with shunts, they will eventually die around post-natal day 30. Animals in this experiment were housed in a cage with their littermates and parents and were maintained in a humidity and temperature-controlled room on a 12 hour light/dark cycle. Food and water were provided *ad libitum*. This model provides a naturally occurring form of non-communicating, obstructive hydrocephalus that correlates with third trimester onset relative to human brain development [26].

### Experimental animal groups

Four groups of animals were examined; n = 5 for all groups:

Group I - Non-hydrocephalic: These rats did not develop hydrocephalus (determined by lack of a domed head on gross examination) and were sacrificed at post-natal day 21. These animals served as the untreated control group.

Group II - Minocycline-treated non-hydrocephalic: These rats were identical to Group I animals except that they received minocycline from day 15 to 21. This comprised the minocycline-treated control group.

Group III - Untreated hydrocephalic: These rats developed severe hydrocephalus (determined as described for Group I) and were left untreated. They were sacrificed at post-natal day 21.

Group IV - Minocycline-treated hydrocephalic: At postnatal day 15, when hydrocephalus is progressing to a severe state [9,27], rats received minocycline treatment for 7 days, ending at sacrifice on day 21 when ventriculomegaly had become severe. Hydrocephalus was again determined using the methods described previously.

### Minocycline Treatment

Due to the small size of the young rats used in this study (20 g body weight vs. 250 g for adults), intravenous injection was not a practical method of minocycline delivery. Therefore, intraperitoneal (IP) injections were used instead. This type of administration has been shown to provide adequate delivery of minocycline to the brain across the blood brain barrier as assessed by both serum and cerebrospinal fluid (CSF) levels [28]. Minocycline-treated animals received minocycline HCl (Sigma Chemicals, St. Louis, USA) dissolved in 5% sucrose at a concentration of 10 mg/ml at a dose of 45 mg/kg/day for 7 days, beginning at postnatal day 15 and ending at day 21 with the termination of the experiment. Injection sites were rotated between the four abdominal quadrants.

### Tissue Processing

At postnatal-day 21, animals were anesthetized with 4% chloral hydrate and perfused transcardially, first with 500 ml of 0.9% saline to flush the vascular system, followed by 500 ml of 4% paraformaldehyde. After perfusion was complete, the brain was carefully and rapidly extracted and post-fixed in 50 ml of 4% paraformaldehyde for 2 h. Brains were then rinsed and stored in buffered saline at 4°C until paraffin embedding. Prior to embedding, hydrocephalic brains were injected with a solution of 4% agar in dH_2_O in order to provide support to the dilated and fragile ventricles. Using an 18-gauge needle and syringe, warm agar was injected through the temporal cortex into the lateral ventricles. The agar-filled brain was then placed into a cool bath of buffered saline, allowing the agar to solidify. All extracted brains from all 3 experimental groups were cut along 2 coronal planes, separating the frontal, parietal, and occipital cortices. Parietal and occipital segments were put in separate, marked embedding trays and placed into the paraffin embedding machine. Tissue was dehydrated in 50, 70, 95, and 100% alcohol, cleared in toluene, and embedded in paraffin wax. Tissue sections were cut in the coronal plane at a thickness of 20 μm as an optimal dimension for stereology (the Optical Fractionator) floated on a warm water bath, and positioned onto glass slides.

### Immunohistochemistry

Sections were de-paraffinized, re-hydrated and incubated in 3% H_2_O_2_. Following procedures described previously [9], astrocytes were detected by the presence of glial fibrillary acidic protein (GFAP); the primary anti-GFAP antibody (DAKO, Carpinteria, USA) was diluted 1:300 and replaced with an anti-rabbit secondary antibody (Vector Laboratories, Burlingame, USA) diluted 1:200. An avidin-biotin complex (ABC kit, Vector) was applied for 30 min and color developed using an un-enhanced diaminobenzidine (DAB) kit (Vector). Slides were dehydrated in increasing concentrations of alcohol, cleared in xylene and cover-slipped with Permount (Fisher Scientific, Pittsburgh, USA). Microglia were detected as described previously [29] using ionized calcium binding adaptor molecule (Iba-1), a marker for both resting and reactive microglia. The same procedures described for GFAP were followed with the exception that primary anti-Iba-1 antibody (Waco, Richmond, VA, USA) was diluted 1: 500.

### Stereological analysis

The full thickness of the cerebral cortex (gray matter laminae and white matter) was examined using light microscopy and stereological quantitative analysis. The observer was blinded to the identity of each animal as well as to the experimental condition as much as possible; because of the obvious thinning of the hydrocephalic cortex it is often impossible to completely blind the analyst. Three regions (2 occipital and 1 parietal) from each brain were selected for analysis.

Glial Density: In order to quantify any glial changes, Stereo Investigator software (MicroBrightField, Inc. Williston, USA) was utilized to obtain an estimated density of both GFAP immunoreactive astrocytes and Iba-1 immunoreactive microglia present in the entire cerebral cortex of treated and untreated hydrocephalic rats. The combination of cell counting using the optical dissector with fractionator sampling is defined as the Optical Fractionator [30,31]. One main benefit of this probe is that data output is not affected by tissue shrinkage which occurs during the tissue preparation process. Using a counting frame of 200 × 200 μm, 45 areas of the neocortex were randomly probed (15 frames per coronal section × 3 sections/animal), marking all immune-labelled cells (the presence of either a soma or process was recorded as a positive occurrence). All probing was performed at 400 × using brightfield optics. By systematically sampling a known fraction of section thickness, along with a known fraction of a sectional area, it was possible to produce an unbiased estimate of the total number of astrocytes and microglia present in a given volume of cortex. Note that this type of sampling included both gray and white matter regions of the cerebral cortex and because of the extreme thinning of white matter in severely hydrocephalic brains, no attempts were made to evaluate these two regions individually. Using the estimated area calculated by the program's planimetry data-generating function, a glial density volume (glial cells/μm^3^) was obtained for each section viewed.

Cortical Thickness: The thickness of the cerebral cortex (pial to ventricular surface) was measured perpendicular to the pial surface using the Stereo Investigator "linear measurement" tool. At 50 × magnification, thickness was measured at the most dorsal portion of the hemisphere, the most lateral portion, and the most inferior portion of the temporal region of each section of brain.

### Statistics

Analysis of glial density was carried out by light microscopic examination of three 20 μm thick coronal sections from each rat, including one parietal and two occipital sections per animal. The mean value of these three sections was determined to yield a representative value of cell density for each animal. Comparisons between groups were determined by performing an ANOVA test followed by a Bonferroni correction. No data were expunged and all values obtained were utilized in the statistical analyses.

Using the same three sections described above from each animal, a slightly different method of data analysis was used for cortical thickness. On each section, three regions of the cortex were measured for thickness. These were the dorsal, lateral, and temporal regions of the brain section, and each was analyzed separately. Data were analyzed for statistical significance by performing an ANOVA test followed by a Bonferroni correction.

## Results

### GFAP- immunoreactive astrocyte number

**Figure 1:**
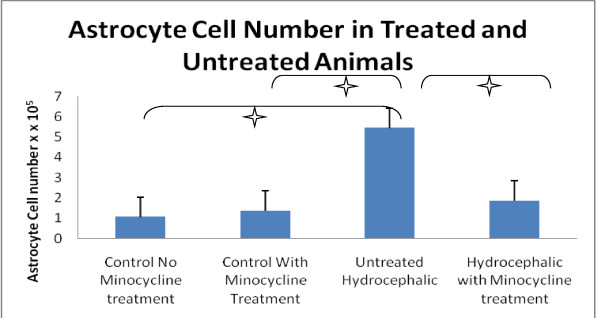
**Astrocyte density (cells/μm^3^) in minocycline treated and untreated animals**. The number of GFAP-immunoreactive cells was significantly higher (stars) in untreated hydrocephalic animals compared to both types of control (*p *< 0.001). Minocycline treatment significantly reduced the number of immunoreactive cells in hydrocephalic animals (*p *< 0.001). No significant differences were found between the control animals receiving minocycline and those that did not receive the drug, suggesting that minocycline has no effect on the normal brain. Data are mean ± SD, n = 5, cell counts were multiplied by 10^5 ^to get the actual cell number per μm^3^.

The number of GFAP-immunoreactive astrocytes was significantly increased in untreated hydrocephalic animals, but minocycline treatment significantly reduced this number (Figure 1). The number of GFAP-immunoreactive astrocytes was significantly higher (*p *< 0.001) in untreated hydrocephalus (mean 5.43 cells/μm^3 ^+/- 2.04 SD) compared to both types of controls: minocycline treated mean 1.35 cells/μm^3 ^+/- 0.28 SD and non-minocycline mean 1.05 cells/μm^3 ^+/- 0.22 SD. A statistically significant decrease in the number of GFAP-immunoreactive astrocytes (*p *< 0.001) occurred in the minocycline-treated hydrocephalic group (mean 1.85 cells/μm^3 ^+/- 0.63 SD) and this number was not significantly different compared to controls. Finally, minocycline treatment alone had no significant effect in control animals. All cell counts were multiplied by 10^-5 ^to obtain the cell number per micrometer squared.

### Iba-1- immunoreactive microglial number

**Figure 2:**
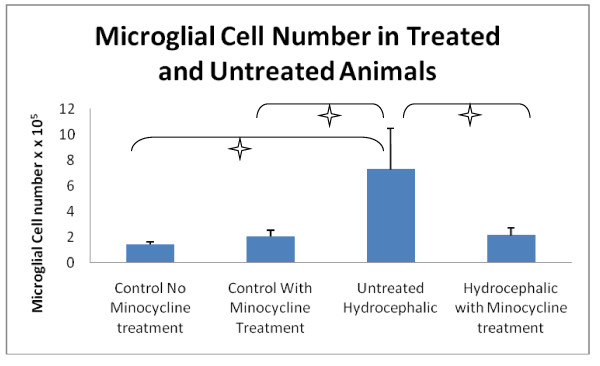
**Microglia density (cells/μm^3^) in minocycline treated and untreated animals**. The number of immunoreactive microglia in untreated hydrocephalic animals was significantly higher than in the minocycline-treated hydrocephalic group (*p *< 0.001). Minocycline treatment had no effect on control brains. There was a statistically significant increase between both control groups compared to the untreated hydrocephalic animals (*p *< 0.001). Data are mean ± SD, n = 5, cell counts were multiplied by 10^-5 ^to get the actual cell number per per μm^3^.

Minocycline treatment significantly reduced the number of Iba-1 immunoreactive microglial cells present in the hydrocephalic brain (Figure 2). Significant differences (*p *< 0.001) were present between the hydrocephalic animals receiving minocycline (mean 2.10 cells/μm^3 ^+/- 0.60 SD) and the untreated hydrocephalic animals (mean 7.27 cells/μm^3 ^+/- 3.21 SD), as well as both control groups. Likewise, the number of Iba-1 immunoreactive microglia in untreated hydrocephalic animals was significantly higher (*p *< 0.001) than in both control animals receiving minocycline (mean 2.00 cells/μm^3 ^+/- 0.50 SD) and control animals not receiving minocycline (mean 1.41 cells/μm^3 ^+/- 0.22 SD). It is important to note that no significant differences were found between the two control groups. All cell counts were multiplied by 10^5 ^to obtain the number of cells per micrometer squared.

### Effect on cortical thickness

Overall, four outcomes were observed with regard to the thickness of the cerebral cortex (Table 1): (1) Untreated hydrocephalic animals exhibited a decreased cortical thickness compared to non-hydrocephalic controls with and without minocycline (* and + symbols in Table 1); this effect was almost always statistically significant. (2) Minocycline treatment in hydrocephalic animals significantly increased cortical thickness compared to untreated hydrocephalic animals in most regions (β symbol in Table 1). (3) This increase restored cortical thickness to control levels in some regions, but six of 9 regions remained significantly thinner compared to controls with and without minocycline (# and δ symbols in Table 1). (4) Finally, minocycline treatment had no effect on cortical thickness in non-hydrocephalic control animals.

**Table 1:** Cortical thickness of all regions (parietal, occipital 1 and occipital 2) and locations (dorsal, lateral and temporal) in the four groups of experimental animals (n = 5 for all groups).

	Mean cortical thickness in mm ± SD			
**Cortical Region**	**Control no minocycline**	**Control with minocycline**	**Untreated hydrocephalic**	**Minocycline-treated hydrocephalic**

***Parietal***				
Dorsal	1.44 ± 0.13	1.32 ± 0.27	0.55 ± 0.27 ^+++^	0.76 ± 0.34 ^ββ, #, δδ^
Lateral	1.78 ± 0.11	1.41 ± 0.14	0.71 ± 0.32	1.12 ± 0.67
Temporal	1.59 ± 0.21	1.79 ± 0.53	0.33 ± 0.16 ^+++^	0.58 ± 0.37 ^βββ, ###, δδδ^

***Occipital 1***				
Dorsal	1.26 ± 0.13	1.25 ± 0.18	0.34 ± 0.09**	0.61 ± 0.15 ^βββ, ###,δδδ^
Lateral	1.29 ± 0.11	1.23 ± 0.12	0.35 ± 0.12 *^, +++^	0.74 ± 0.28
Temporal	1.24 ± 0.14	1.17 ± 0.30	0.21 ± 0.12 **^, +++^	0.59 ± 0.24

***Occipital 2***				
Dorsal	2.04 ± 0.27	1.04 ± 0.15	0.33 ± 0.09 *^, +++^	0.45 ± 0.11 ^ββ, ##, δδ^
Lateral	3.60 ± 0.78	1.29 ± 0.42	0.28 ± 0.14 ^+++^	0.48 ± 0.24 ^βββ, ###, δδδ^
Temporal	3.47 ± 0.31	1.31 ± 0.64	0.31 ± 0.11 ^++^	0.48 ± 0.23 ^ββ, ^^###, δδ^

*, **, *p *< 0.05 and 0.01; Untreated hydrocephalic compared to control no minocycline.

++, +++, *p *< 0.01 and 0.001; Untreated hydrocephalics compared to control with minocycline.

ββ, βββ, *p *< 0.01 and 0.001; Minocycline-treated hydrocephalic compared to untreated hydrocephalic.

#, ##, ###, *p *< 0.05, 0.01 and 0.001; Minocycline-treated hydrocephalic compared to control no minocycline.

δ δ, δ δ δ, *p *< 0.01 and 0.001; Minocycline-treated hydrocephalic compared to control with minocycline.

## Discussion

The major findings of this study are that systemic treatment with minocycline (1) significantly reduced the numbers of cortical GFAP immunoreactive astrocytes and Iba-1 immunoreactive microglia in hydrocephalic brains, (2) partially restored cortical mantle thickness to control levels, and (3) had no effect on control brains. These effects are impressive because in the cortex of untreated hydrocephalic brains the numbers of GFAP immunoreactive astrocytes and Iba-1 immunoreactive microglia increase significantly about 5-fold and the cerebral cortex is reduced to about 1/3 of its normal thickness. From a clinical perspective, these results are interesting because minocycline was administered during relatively late stages of ventriculomegaly, and thus the treatment effects apply specifically to astrocytes and microglia in advanced phases of reactivity.

### Role of glia in the pathophysiology of hydrocephalus

In hydrocephalus, gliosis is known to occur, especially in the periventricular white matter [4,5,7,32-36] but also in cortical gray matter [10]. Glial scar formation could play a major role in creating the problems that chronically plague hydrocephalic children. It has been suggested by many investigators [37], including ourselves [10], that gliosis is a permanent fixture in hydrocephalic brains, even those that have been shunted successfully. Our previous studies have shown that GFAP RNA levels increase with the progression of hydrocephalus in both a congenital model of rodent hydrocephalus (the H-Tx rat) [9] and a kaolin model of induced hydrocephalus in kittens [8]. Furthermore, Yoshida found that GFAP-labeled reactive astrocytes were present surrounding cystic lesions in severely hydrocephalic H-Tx animals, but was not able to detect a significant increase in GFAP labeled astrocytes in the white matter surrounding the ventricles [38,39]. Clinically, increased levels of GFAP protein have been found in the CSF of patients with normal pressure hydrocephalus, and patients who developed secondary hydrocephalus due to subarachnoid hemorrhage [40-43], and the possibility of using GFAP protein levels as a diagnostic tool for hydrocephalus is currently being explored [44,45]. Finally, previous studies in our laboratory on a kitten model of kaolin-induced hydrocephalus demonstrated that shunting could reduce the amount of GFAP protein and RNA present in the cerebral cortex, but the results were quite variable and GFAP levels began to rise over time (unpublished observations).

Microglia play a major role in the response to brain injury (reviewed in [46-50]), including hydrocephalus [9,10,51-54]. Mangano *et al *[10] illustrated that microglial cell proliferation and activation increased in regions of the sensorimotor cortex and auditory cortex during the progression of hydrocephalus in moderately affected H-Tx rats. When assessing microglial changes in pediatric hydrocephalus, it is important to remember that normal microglia change morphology during differentiation. The typical maturation process of a microglial cell in rats has been demonstrated impressively by Orlowski *et al*. [55], and these features were used for maturation comparisons in the current study. Immature microglia have an amoeboid shape with large somata and very few short processes with growth-cone-like varicosities, but as these cells mature cellular processes become longer and more numerous and by postnatal day 30 in rats, cells have acquired the fully ramified fine long processes with extensive branching typical of resting microglia.

It is also important to note that while the present study involved counts of GFAP- and Iba-1-immunoreactive cells, the analysis does not measure proliferation of astrocytes and microglia directly. Since it is possible that less reactive cells expressing low or undetectable levels of GFAP and Iba-1 would not be counted, a more definitive labeling study with BrdU or Ki67 should be performed to assess cellular proliferation.

### The role of minocycline

Minocycline, a derivative of the well-known antibiotic tetracycline, has recently shown promise as a specific inhibitor of microglial cells, one of the main elements of glial scar formation in hydrocephalus. Although its mechanism of action is still unknown [18,56-60], minocycline's promise as a neuroprotective agent is illustrated by the recent initiation of clinical trials in Parkinson's disease [61]. Since its initial demonstration as an anti-inflammatory agent [62], minocycline has been shown to have multiple benefits in brain injury (reviewed in Buller *et al *[18]). Minocycline can inhibit microglial activation, prevent glutamate toxicity, prevent caspase-1 and caspase-3 activated apoptosis, decrease the activity of inducible nitric oxide synthetase and p-38 mitogen activated protein kinase, inhibit matrix metalloproteinase-2 activity, and impair cytokine production. Additionally, the mechanism of action for minocycline involves not only microglia directly but also T cells and their subsequent activation of microglia. These actions of minocycline appear to be responsible for reducing brain injury in animal models of Parkinson's disease, Huntington's disease, amyotrophic lateral sclerosis, traumatic brain injury, excitotoxicity, intracerebral hemorrhage, spinal cord injury, focal and global cerebral ischemia, and hypoxia.

### The potential use of minocycline in hydrocephalus

To date, one study has attempted to protect the hydrocephalic brain by infusion of nimodipine, a calcium channel antagonist, into the ventricles of juvenile hydrocephalic rats in order to reduce white matter damage. Unfortunately, these interventions have produced only limited short-term success [63-65]. Another study has demonstrated that GFAP can be reduced in the hydrocephalic brain by administration of magnesium sulphate [66]. Thus the current study represents a relatively novel effort that, based on the previous successes achieved in other brain injury models, has a strong likelihood of promoting supplemental treatments for hydrocephalus.

One important caveat is worth noting: tetracycline (from which minocycline is a derivative) administration can impair bone and tooth development, as well as cause discoloration of teeth [18] in children. Whether minocycline also exerts these effects is not known, but this potential problem must be taken into consideration before clinical applications can be adopted.

## Conclusions

Our data suggest that minocycline treatment is effective in reducing the gliosis that accompanies ventriculomegaly, and thus may provide an added benefit when used as a supplement to ventricular shunting. With additional pre-clinical data, one would anticipate that this new avenue of research would eventually culminate in a clinical study using minocycline as a supplemental treatment to shunting in human patients.

## Competing interests

The authors declare that they have no competing interests.

## Authors' contributions

JMM participated in the design of the study, carried out the histologic work, supervised technicians in the laboratory helping with histology, gathered all data, performed the statistical analysis and helped to draft the manuscript. JPM participated in the design of the study, acquired funding for the project, and helped to draft the manuscript. All authors read and approved the final manuscript.
